# Determination of Physicomechanical Characteristics of the Cement Mortar with Added Fiberglass Waste Treated with Hydrogen Plasma

**DOI:** 10.3390/ma14071718

**Published:** 2021-03-31

**Authors:** Marius Lucian Lupu, Dorina Nicolina Isopescu, Ioan Tuns, Ioana-Roxana Baciu, Sebastian George Maxineasa

**Affiliations:** 1 Department of Civil and Industrial Engineering, Gheorghe Asachi Technical University of Iasi-Romania, Blvd. Mangeron, No. 1, 700050 Iasi, Romania; mariuslucianlupu@gmail.com; 2 Department of Construction, The Transilvania University of Brasov-Romania, Tower Street, No. 5, 500152 Brașov, Romania; ioan.tuns@unitbv.ro

**Keywords:** fiberglass, hydrogen, plasma, cement, mortar, vitrification

## Abstract

Solving the environmental problems and the economic aspects of the construction sector represent a global priority. The considerable quantities of raw materials and the energy consumed by this sector make it one of the most polluting economic activities. Fiberglass in various forms is widely used in the construction sector. In the manufacturing process and during the usage of fiberglass products, a significant amount of indestructible waste results, negatively impacting the environment. An innovative solution for utilizing this type of waste is the treatment with hydrogen plasma. This process results in two products: the first in the gaseous state used to obtain synthetic fuel and the second in solid-state, named slag. The composition of solid waste contains chemical compounds that can increase their strength if used as additives in mortars or concretes. This study presents the laboratory tests on mortars, in which a part of the cement amount was replaced with the solid component resulting from the plasma treatment of glass fiber waste. The results showed that replacing a part of the cement with these materials is a solution that minimizes the ecological footprint of the buildings.

## 1. Introduction

In the current circumstances, where there is an increasing question of the negative influence humanity has on the environment, effective municipal waste management is a hotly debated topic, internationally. It is known that the construction sector is responsible for the consumption of a significant amount of raw materials and non-renewable energy, which positions it as one of the most polluting economic activities globally. The construction sector has a high impact on a community, ecologically, socially, and economically [1,2]. Since the construction materials industry is of great interest in using waste in producing new materials, the new categories of materials are developing in line with sustainability policies [3,4].

One of the most used materials in the construction sector is concrete. Concrete is a mixture of natural aggregates, cement, and water, making it easy to produce anywhere. Concrete currently contains approximately 12% cement, 8% water, and 80% aggregate in mass. Aggregates and water come from natural resources, while cement is a manufactured product. Its production process has a significant negative impact on the environment (for each kilogram of cement produced, approximately one kilogram of CO_2_ equivalent is released into the atmosphere) [5,6]. 

In line with the steps to protect the environment and reduce pollution globally, various replacement materials, or the reduction of aggregate and cement use, have been studied over time. In the specialty literature, several studies were conducted that were mainly aimed at the use of various wastes in the construction sector, thus noting the study on cement mortar containing oil-contaminated sand, which presents the characteristics, strength development, and microstructure of this type of mortar [7].

**Figure 1:**
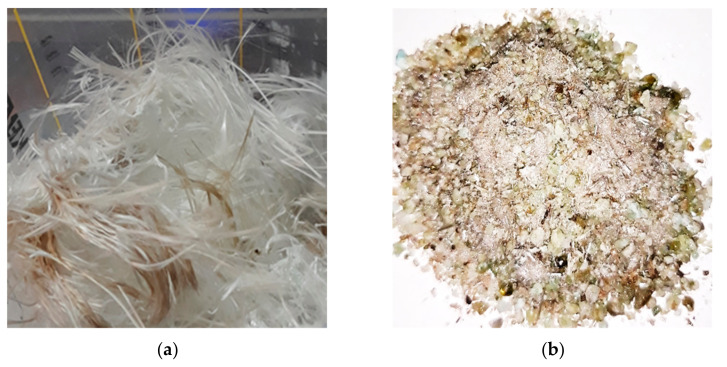
(**a**) Fiberglass waste; (**b**) fiberglass waste vitrified with hydrogen plasma.

Another commonly used material in the construction sector is fiberglass (Figure 1a). Fiberglass is predominantly used in the form of glass wool (as a heat-insulating and pho-no-insulating material), or fiberglass mesh (for reinforcement of sheets or plasters). Even though fiberglass has many benefits, it does not biodegrade, and recycling solutions are needed. When fiberglass products are manufactured, and after their use, a large amount of waste is not biodegradable and has a considerable impact on the environment [8].

The waste management process, in general, involves the treatment of waste in different forms of aggregation. The selection of treatment has, as essential input data, the characteristics of waste biodegradability. A variety of waste recycling solutions can be used today in this process, these being extended on a large scale by the circular economy principles implementation. In the construction sector, waste is primarily solid and non-biodegradable, having a significant impact on the environment. Thus, waste management processes and methods are needed to minimize the environmental impact. One of the most recent and most effective methods of waste treatment is hydrogen plasma conversion.

The plasma conversion system transforms any waste using a neutral gas, hydrogen in our case, through which a strong electromagnetic field passes until it becomes ionized, increasing its electrical conductivity [9]. It is essential to note that it is not about incineration but dissociation at a molecular level in this system. When a substance is subjected to molecular dissociation, it is not only changing its condition, but disintegrates. At this point, it is no longer “a substance”; all that remains are the component atoms and the decimated molecules. When the molecules are subjected to intense energies (plasma torch), the bonds that hold the molecules are broken [10,11]. What remains are the component elements of the molecules. Dangerous substances such as cyanide, for example, results in carbon and nitrogen atoms. Organic compounds are volatile and converted into synthetic gas (syngas), which can be used as a fuel source if purified. Inorganic compounds are melted and converted into a vitrified rock containing metal in addition to other inorganic substances, such as slag [9,10,11].

Depending on the type of waste material, the plasma treatment can consist of two phenomena: gasification and vitrification [12,13]. Gasification is a process that transforms carbon-containing materials such as coal, petroleum coke, municipal solid waste, or biomass into a synthesis gas (syngas), composed mainly of carbon monoxide and hydrogen [13,14]. Vitrification is the process by which silicates, or a mixture of silicates, are transformed into a glassy amorphous mass following exposure to high temperatures, resulting in melting followed by solidification [15,16].

Research has led to the use of grinding, incineration, and pyrolysis for the recycling of fiberglass. Fiberglass finds its way into different industries and can be used in various end products [10]. For example, fiberglass reinforcement effectively reduces concrete shrinkage, thereby increasing its durability [17]. This concrete can best be used in frozen temperate areas for floors, sidewalks, and concrete curbs [18,19,20].

In the context of the sustainable development of the construction sector, this research studies the use of hydrogen plasma-treated fiberglass waste (Figure 1b) and its incorporation into cement mortars, particularly looking at the influence that these additional materials have on the mechanical characteristics of mortar. 

This study presents the laboratory tests on mortars in which a part of the cement amount was replaced with the solid component resulting from the plasma treatment of glass fiber waste. In the research regarding the replacement of cement with other substances to diminish the negative effect of this material on the environment, a limitation of 20–25 wt %, is accepted. Considering the novelty of the research presented in this article, it was initially aimed at replacing cement in smaller percentages, namely 3 wt %, 6 wt %, and 10 wt %. For the beginning, these percentages were chosen, and if the results were favorable, the research would have continued, taking into account higher percentages.

The obtained results showed replacing a part of the cement with these material additions is a solution that minimizes the ecological footprint of the buildings in terms of the environmental impact. The results obtained by replacing a small amount of cement (3 wt %; 6 wt %; 10 wt %) highlighted that the solid component obtained from the plasma treatment of glass fiber waste, which has in the chemical component Si and Ca, has a positive influence on the mortar resistance. These chemical compounds are complementary to the formation of hydration products of cement, respectively, for the calcium silicates.

## 2. Materials and Methods

### 2.1. Analysis of Fiberglass Waste (before and after the Hydrogen Plasma Treatment)—Scanning Electron Microscopy (SEM) and Energy Dispersive X-Ray (EDX) Analysis

Determination of fiberglass waste characteristics before and after the hydrogen plasma process was made with the QUANTA 200 3D electron microscope [21]. First of all, a scanning electron microscope (SEM) (FEI, Brno, Czech Republic) analysis was conducted, which presents a focused electron beam over a surface to create an image. The electrons in the beam interact with the sample, producing various signals that can be used to obtain information about the surface topography and composition. Secondly, there were carried out energy dispersive X-ray analysis (EDX) (FEI, Brno, Czech Republic), an X-ray technique used to identify materials’ elemental composition. This analysis is applicable in materials and product research [22,23,24].

**Figure 2:**
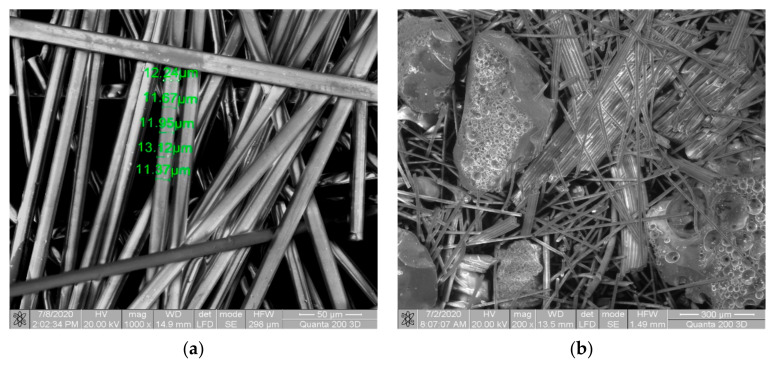
SEM analysis for (**a**) fiberglass waste; (**b**) fiberglass waste treated with hydrogen plasma.

In the SEM analysis of the fiberglass waste can be observed the dimensions of the fibers between 11.9–13.12 μm, and for the fiberglass waste treated with hydrogen plasma, the analysis presents glomerules and irregularities resulting from the treatment with hydrogen plasma (Figure 2).

**Figure 3:**
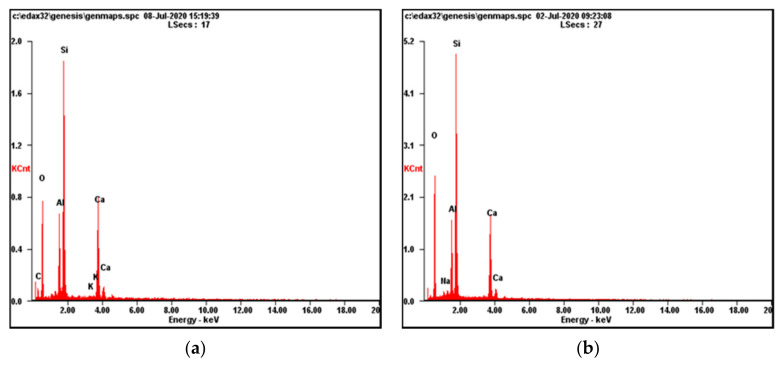
EDX analysis for (**a**) fiberglass waste; (**b**) fiberglass waste treated with hydrogen plasma.

EDX analysis indicated the presence of a silicon (Si) peak at 1.9 keV for both fiberglass waste and fiberglass waste treated with hydrogen plasma. The difference is the value of the silicon peak, noting that in the case of the treated fiberglass waste, the value is approximately 5.1 KCnt. In contrast, untreated fiberglass waste is about 1.9 KCnt (Figure 3). It can also be observed that in the case of untreated fiberglass waste, carbon (C) and potassium (K) are present. After the hydrogen plasma treatment, these two chemical elements disappear, being replaced by sodium (Na). 

Recent studies show that sodium silicate permeates into concrete, which favors the reaction with portlandite in the cement matrix to yield calcium–silicate hydrates. This reaction blocks the concrete pores, increases its surface hardness, and structural impermeability, and durability. The investigations based on these substances did not reveal the exact mechanism by which the concrete performance was improved [25,26,27,28].

**Figure 4:**
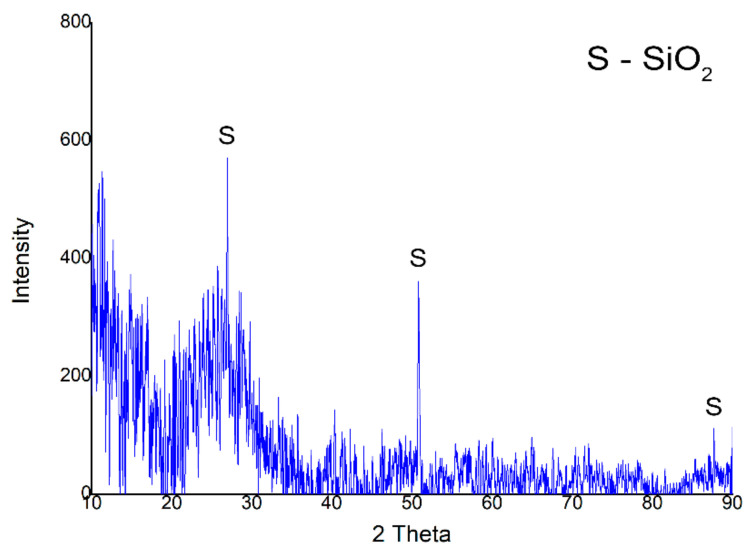
XRD pattern of fiberglass waste treated with hydrogen plasma.

Further X-ray diffraction (XRD) (PANalytical, Almelo, Netherlands) analysis on the fiberglass waste treated with hydrogen plasma sample revealed SiO_2_ peaks (COD 96-153-8065), the major peak 26.64°, and the minor peak at 51.15°, having a hexagonal crystalline structure. The XRD analysis results (Figure 4) are consistent with EDX analysis, in which the main elements are Si and O.

### 2.2. Experiment Description—Determination of Flexural and Compressive Strength

Flexural and compressive strength were determined under SR EN 196-1 with prismatic test samples with a size of 40 mm × 40 mm × 160 mm. The sand used is standardized C.E.N., according to EN 196-1, each prepackaged sandbag weighing 1350 ± 5 g [29]. The cement used is CEM II/B-LL 42.5 N. Tap water from the public network was used to carry out the samples. 

The proportions by mass for the control samples (PM) are one part cement, three parts standardized sand C.E.N. and half part water, water/cement ratio of 0.5 [29].

Each mixture of three control samples (PM) consists of [29]:

450 ± 2 g cement
1350 ± 5 g standardized sand CEN
225 ± 1 g water

Starting from the above proportions, various percentages of cement were replaced to develop new materials that incorporate products resulting from the hydrogen plasma conversion of fiberglass waste as follows (recipes are for three samples type):

- PS 3 wt %—436.5 ± 1 g cement
  13.5 ± 0.5 g hydrogen plasma vitrified fiberglass
  1350 ± 5 g standardized sand CEN
  225 ± 1 g water
- PS 6 wt %—423 ± 1 g cement
  27 ± 0.5 g hydrogen plasma vitrified fiberglass
  1350 ± 5 g standardized sand CEN
  225 ± 1 g water
- PS 10 wt %—405 ± 1 g cement
  45 ± 0.5 g hydrogen plasma vitrified fiberglass
  1350 ± 5 g standardized sand CEN
  225 ± 1 g water

The mortar was prepared by mechanical mixing and compacted into a mold. The molded samples were stored in a humid atmosphere (relative humidity of 70%) for 24 h, then demolded to be immersed in water until resistance testing. At the optimum time, samples were taken from the water, subjected to bending until breaking, resulting in two halves, and half being tested at compression [29]. The machine used to determine flexural and compressive strength is a hydraulic universal testing machine, type WAW-600E.

The testing term of the prisms has been calculated from the moment they were molded with the following limits [29]:

- 2 days ± 30 min.
- 7 days ± 30 min.
- 28 days ± 30 min.

All test procedures complied with the provisions of SR EN 196-1 [29].

Flexural strength, R_f_, was determined according to Equation (1) [29]:

R_f_ = 1.5 × F_f_ × l/b^3^ (1)

where R_f_ represents the flexural strength (MPa), associated with the side of the square section of prism b (mm), the load applied to the middle of the prism at fracture F_f_ (N), and the distance between the supports, l (mm) [29]. 

Compressive strength, R_c_, was evaluated according to Equation (2) [29]:

R_c_ = F_c_/A_c_ (2)

where R_c_ represents the compressive strength (MPa), associated with the maximum load at fracture F_c_ (N), and the cross-sectional area of the specimen on which the compressive force acts (1600 mm^2^) [29].

## 3. Results

### 3.1. Determination of Flexural Strength

The flexural strength test’s average value was calculated as the arithmetic means of the three individual results [29].

Table 1 presents the control sample’s flexural strength results and the three hydrogen plasma vitrified fiberglass samples tested (PM, P.S.3 wt %, P.S.6 wt %, P.S.10 wt %), their fracture load, and the standard deviation for all three ages of curing (2, 7, and 28 days).

**Figure 5:**
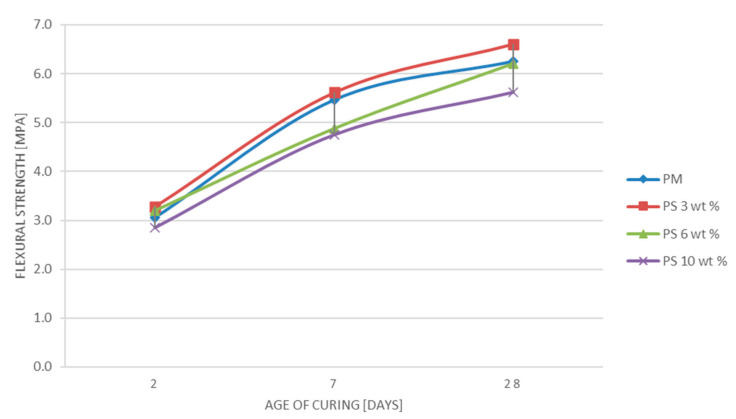
The diagrams of flexural strength results for samples with hydrogen plasma vitrified fiberglass (PS 3 wt %, PS 6 wt %, and PS 10 wt %) and the control samples (PM).

In Figure 5 are presented the diagrams of the flexural strength results for samples with hydrogen plasma vitrified fiberglass (PS 3 wt %, PS 6 wt %, and PS 10 wt %), and the control sample for the three tested periods (2 days, 7 days, and 28 days), highlighting the behavior of each batch of samples and their evolution over time.

### 3.2. Determination of Compression Strength

Compression strength test results were calculated as the arithmetic means of the six individual results [29].

**Figure 6:**
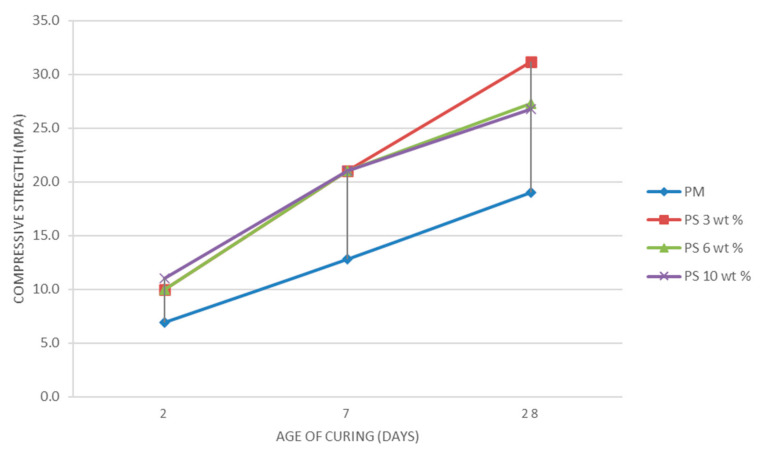
The diagrams of compression strength results for samples with hydrogen plasma vitrified fiberglass (PS 3 wt %, PS 6 wt %, and PS 10 wt %) and the control sample (PM).

Table 2 presents the control sample’s compression strength results and the six hydrogen plasma vitrified fiberglass samples tested (PM, PS 3 wt %, PS 6 wt %, PS 10 wt %), their fracture load, and the standard deviation for all three ages of curing (2, 7, and 28 days). The compression strength results for samples with hydrogen plasma vitrified fiberglass (PS 3 wt %, PS 6 wt %, and PS 10 wt %) and the control sample for the three test periods (2 days, 7 days, and 28 days), highlighting the average behavior of each batch of samples and their evolution over time are presented in Figure 6.

## 4. Discussion

The presented research’s primary goals are to analyze, develop, and optimize different solutions to significantly reduce the construction sector’s negative influence on the natural environment. At the same time, new plasma technologies can turn waste into raw materials, contributing to environmental protection in large urban areas. In the technical literature, the research on reducing cement consumption in mortars and concretes by replacing a part of it with by-products (ultrafine silica, power plant ash, slag, etc.) has experienced a growing trend. The studies have been carried out to ensure that new products developed (mortars or concretes that comply with standardized strength class with different composition) have strengths and rigidity characteristics applicable in the construction sector [30,31,32,33,34].

Following the tests carried out, the average values obtained in the case of the flexural strength of the mortar specimens underlined the fact that the reduction of cement by 3 wt % and its replacement with products resulting from fiberglass waste treated with hydrogen plasma improves the flexural strength. The values obtained for the PS 3 wt % specimens show an improvement, with approximately 5% compared to the control specimens, PM, for all tested curing times, given by standards [29].

The average values of flexural strength, in the cases of reductions of 6 wt % and 10 wt %, the PS 6 wt % and PS 10 wt % samples, are lower than those of the control specimens; therefore, it can be stated that, after these tests, there is no certainty of obtaining mortars comparable, in terms of flexural strength, with control mortars.

For the mortars’ compressive strengths, the results obtained in all studied cases reveal that the average values are higher than the average value of the control samples’ compressive strengths. The highest increase is also obtained in the case of PS 3 wt % samples.

## 5. Conclusions

The research presented in the study showed that a quantity of cement could be replaced with products resulting from fiberglass waste treated with hydrogen plasma. In construction, structural elements are rarely subjected to compression forces only. Most often, they are subjected to bending or, to bending and compression. Therefore, a limit in the amount of replaced cement by fiberglass waste treated with hydrogen plasma should be considered 3 wt %.

The experiment results showed that the particles of fiberglass vitrified with plasma hydrogen increased the viscosity, and slightly improved the mortars’ strength, due to their pozzolanic activity. The added particles led to the quantitative increase of the hydration products, and thus enhanced the value of the compressive strength of the mortars due to the supplementary formation of a calcium-silicon hydrated compound, which strengthened the microstructure of the mortars. In addition, in the case of compressive strength of mortars, a positive influence was due to the larger particles added that consolidated the mortar’s structure. 

The tests for flexural strengths have shown that the addition of fiberglass waste treated with hydrogen plasma in percentages higher than 3 wt % can decrease the resistances due to the granularity of the mineral addition. Even if the mortar’s compactness is better, the volume of included air increased, thus causing a decrease in the mortar’s tensile strength.

By replacing a quantity of cement with fiberglass waste treated with hydrogen plasma, in terms of mechanical characteristics, mortars similar to the usual ones can be obtained, having advantages from an economic and ecological perspective. The results obtained are promising and underline the necessity for continuing the research regarding the strength and rigidity characteristics in conditions of the static and dynamic loads, and life-cycle assessment analysis of these materials. It is observed that the replacement of cement with the complementary product analyzed in the paper, within certain limits, leads to improvement of resistances. Based on the research carried out and presented in this article, a new research direction can be established using different additives to increase the workability and reduce the water/cement ratio.

## Figures and Tables

**Table 1:** Flexural strength (*R_f_*), fracture load (*F_f_*), and the standard deviation (*SD*) results for the control sample and the hydrogen plasma vitrified fiberglass samples tested (PM, PS 3 wt %, PS 6 wt %, and PS 10 wt %).

	Samples	Age of Curing					
		2 Days	SD	7 Days	SD	28 Days	SD
Fracture load—F_f_ [kN]	PM	1.31	-	2.33	-	2.63	-
	PS 3 wt %	1.40	-	2.40	-	2.82	-
	PS 6 wt %	1.36	-	2.08	-	2.65	-
	PS 10 wt %	1.21	-	2.03	-	2.44	-
Flexural strength—R_f_ [MPa]	PM	3.1	0.27	5.5	0.05	6.3	0.41
	PS 3 wt %	3.3	0.12	5.6	0.56	6.6	0.21
	PS 6 wt %	3.2	0.23	4.9	0.37	6.2	0.18
	PS 10 wt %	2.8	0.21	4.8	0.37	5.6	0.49

**Table 2:** Compression strength (*R_c_*), fracture load (*F_f_*), and the standard deviation (SD) results for the control sample and the hydrogen plasma vitrified fiberglass samples tested (PM, PS 3 wt %, PS 6 wt %, and PS 10 wt %).

	Samples	Age of Curing					
		2 Days	SD	7 Days	SD	28 Days	SD
Fracture load—F_c_ [kN]	PM	11.08	-	20.48	-	30.84	-
	PS 3 wt %	16.01	-	33.55	-	49.95	-
	PS 6 wt %	15.95	-	33.67	-	43.64	-
	PS 10 wt %	17.54	-	33.58	-	43.25	-
Compression strength—R_c_ [MPa]	PM	6.90	0.52	12.8	0.47	19.0	0.91
	PS 3 wt %	10.0	0.65	21.0	0.94	31.2	0.98
	PS 6 wt %	10.0	0.32	21.0	0.84	27.3	0.80
	PS 10 wt %	11.0	0.57	21.0	0.76	26.8	0.67

## Data Availability

Data sharing not applicable.
